# Discontinuation of tuberculosis treatment among children in the Kampala Capital City Authority health facilities: a mixed-methods study

**DOI:** 10.1186/s12879-021-06244-y

**Published:** 2021-06-01

**Authors:** Leonard Kibirige, Jonathan Izudi, Stephen Okoboi

**Affiliations:** 1 Institute of Public Health and Management, Clarke International University, P.O. Box 7782, Kampala, Uganda; 2 Department of Community Health, Faculty of Medicine, Mbarara University of Science and Technology, P.O. Box 1410, Mbarara, Uganda; 3 Infectious Diseases Institute, School of Medicine, Makerere University College of Health Sciences, P.O. Box 22418, Kampala, Uganda

**Keywords:** Childhood tuberculosis, Tuberculosis treatment discontinuation, Children with tuberculosis, Uganda

## Abstract

**Introduction:**

Discontinuation of tuberculosis treatment (DTT) among children in sub-Saharan Africa is a major obstacle to effective tuberculosis (TB) control and has the potential to worsen the emergence of multi-drug resistant TB and death. DTT in children is understudied in Uganda. We examined the level and factors associated with DTT among children at four large health facilities in Kampala Capital City Authority and documented the reasons for DTT from treatment supporters and healthcare provider perspectives.

**Methods:**

We conducted a retrospective analysis of records for children < 15 years diagnosed and treated for TB between January 2018 and December 2019. We held focus group discussions with treatment supporters and key informant interviews with healthcare providers. We defined DTT as the stoppage of TB treatment for 30 or more consecutive days. We used a stepwise generalized linear model to assess factors independently associated with DTT and content analysis for the qualitative data reported using sub-themes.

**Results:**

Of 312 participants enrolled, 35 (11.2%) had discontinued TB treatment. The reasons for DTT included lack of privacy at healthcare facilities for children with TB and their treatment supporters, the disappearance of TB symptoms following treatment initiation, poor implementation of the community-based directly observed therapy short-course (CB-DOTS) strategy, insufficient funding to the TB program, and frequent stock-outs of TB drugs. DTT was more likely during the continuation phase of TB treatment compared to the intensive phase (Adjusted odds ratio (aOR), 5.22; 95% Confidence Interval (CI), 1.76–17.52) and when the treatment supporter was employed compared to when the treatment supporter was unemployed (aOR, 3.60; 95% CI, 1.34–11.38).

**Conclusion:**

Many children with TB discontinue TB treatment and this might exacerbate TB morbidity and mortality. To mitigate DTT, healthcare providers should ensure children with TB and their treatment supporters are accorded privacy during service provision and provide more information about TB symptom resolution and treatment duration versus the need to complete treatment. The district and national TB control programs should address gaps in funding to TB care, the supply of TB drugs, and the implementation of the CB-DOTS strategy.

## Introduction

Tuberculosis (TB) remains one of the leading causes of death among children living with human immunodeficiency virus (HIV) and a major cause of antimicrobial resistance [1–3]. Global estimates indicated that 10 million people are newly diagnosed with TB disease on yearly basis, of which 1.2 million are children below 15 years of age. Nearly 650 children with TB die daily and four in every five of the deaths occur before the fifth birthday [4]. About a quarter of the global TB burden is in sub-Saharan Africa, mainly among children with TB/HIV [2]. The protection of children against TB is a cornerstone of social and family culture [5].

In Uganda, it is estimated that the annual incidence of bacteriologically confirmed pulmonary TB, both new and relapse cases, notified in 2019 is nearly 65,900 and the incidence of TB in all forms stands at 200 per 100,000 population [6]. Compared to adults with TB, in children, TB is associated with faster disease progression, severe and complicated forms of TB, and a higher risk of death [7]. Accordingly, prompt diagnosis, early treatment initiation, and treatment complication are important in preventing poor prognosis [7]. Discontinuation of TB treatment (DTT) among children remains a global public health problem [8] and is worst in the sub-Saharan Africa region (1). Several factors are associated with non-adherence and loss to follow-up among TB patients including children in developing countries leading to DTT among children and include among others, long treatment durations, high pill burden, medication-related side-effects, and symptom resolution [9]. DTT thus remains a major obstacle to efficient TB control in developing countries like Uganda and has the potential to worsen the emergence of multi-drug resistant TB and death [10, 11].

Some of the notable approaches to tackling DTT in children include the development of child-friendly policies, an integrated, family-based approach to TB care and services, addressing vulnerabilities faced by children with TB, providing support to their treatment supporters particularly women and the elderly, providing them with social protection, and promoting equitable access to child-friendly formulations of medicines to optimize treatment [12]. Other important approaches to preventing loss-to-follow up and non-adherence aimed at reducing DTT in children include committing to tracking the lost to follow-up children with TB, integrating TB efforts fully into relevant health services [9].

Across the Kampala Capital City Authority (KCCA) health facilities, the review of program data show that the majority of children with TB do not complete the entire 6-months of TB treatment, and data about DTT are limited despite being important in strengthening TB follow-up and retention and improving treatment completion rate. Therefore, we examined the level and factors associated with DTT among children with TB across four purposively selected KCCA health facilities and documented reasons for DTT from the treatment supporter and healthcare provider perspectives.

## Methods and materials

### Study design and setting

This retrospective study employed both quantitative and qualitative approaches and was conducted at four large TB diagnostic and treatment units in the KCCA between January 2018 and December 2019. The study sites included two health center fours (HC IVs) namely Kisenyi and Kawaala, and two HC IIIs thus Kisugu and Kitebi.

A HC IV is equivalent to a county-level health facility and serves a population of over 100,000 people while a HC III is a parish-level health facility and serves a population of over 40,000 people according to Uganda’s health system. The four study sites are located in the four divisions of Kampala which include; Central, Nakawa, Rubaga, and Makindye divisions. All these study sites provide TB diagnostic, health education, and TB counselling on daily basis, and capture data through the Ministry of Health TB unit registers, and provide TB treatment following the national guidelines. Children below 24 kg (Kgs) are treated with a 6-month anti-TB regimen that consists of 2 months of Isoniazid (I), Rifampicin (R), and Pyrazinamide (Z) as a fixed-dose combination and Ethambutol (E) as an independent tablet in the intensive phase and 4 months of HR in the continuation phase, shortened as 2RHZE, or the 8 months regime that consists of 2 months of RHZE and 6 months of HE abbreviated as 2RHZE/6HE before August 2017 [13]. However, as children approach a body weight of 25 Kgs, the adult doses of anti-TB regimen, a fixed-dose combination of 2RHZE and 4RH is used. For children with TB meningitis or bone TB, treatment lasts for 12 months thus 2 months of RHZE and 10 months of RH, shortened as 2RHZE/10RH.

### Study population and sample size

The study population for the quantitative component included the records of all children < 15 years with or without HIV infection, initiated on TB treatment between January 2018 and December 2019. We excluded children with a documented transfer-out status because it was not logistically possible to obtain data about their treatment status. Furthermore, we excluded records for children documented to have multidrug-resistant TB because their treatment takes longer than 6 months, children transferred in but lacked sufficient clinical and treatment histories such as date of treatment initiation, records with missing age information, and children documented to have died.

No sample size was calculated because data were abstracted for a defined period and cohort of children. For the qualitative component, the study population included treatment supporters documented in the TB unit register as caregivers of children with TB and healthcare providers and the sample sizes depended on saturation [14]. Saturation was considered reached when further interviewing of the participants yielded no new information. The treatment supporters were selected purposively on TB clinic days and they were 38 participants in total (8 in the first FGD, 10 in the second FGD, 11 in the third FGD, and 9 in the fourth FGD). A treatment supporter was eligible for the FGD if he/she has been recorded in the TB unit register as the formal caregiver to the child with TB and if he/she was willing to participate in the discussion. We also purposively selected eight health workers within the TB clinic as key informants.

### Data collection

Quantitative data were abstracted from the TB unit registers using a standardized data abstraction tool between May and August 2020. The qualitative data were collected through four focus group discussions (FGDs), each consisting of 8–12 treatment supports and key informant interviews with dedicated TB clinic healthcare workers. The FGDs were held within the premises of the health facility in the local language, “Luganda” spoken by the participants, by two research assistants, KA and OB who were trained in qualitative data collection skills and responsible conduct of research. The FGDs were moderated by KL while KA audio-recorded the sessions, took field notes, and probed whenever necessary. On average each FGD lasted for 40–60 min and all the participants were encouraged to ask questions irrespective of the correctness. Some FDG members’ dominance was handled by asking direct probing questions and comments to members who were engaging less. For key informant interviews (KIIs), two healthcare providers namely, one TB focal person and one nursing officer who provide TB treatment at the respective study sites were purposively selected and interviewed to provide expert opinions concerning DTT.

Each key informant interview lasted for 30–45 min, also conducted within the health facility premises in the English language, in a quiet and convenient room. The FGDs and KIIs were conducted until saturation was achieved [14].

### Study variables

The dependent variable was DTT, defined as the stoppage of TB treatment for 30 or more consecutive days. The independent variables that were examined included sex, age of the child, type of person with TB namely new or previously treated, TB disease classification, type of directly observed therapy short-course (DOTS) strategy, anti-TB regimen prescribed, TB/HIV comorbidity, residence, referral, and phase of TB treatment. The treatment supporter variables examined included marital status, employment status, relationship with the child with TB, and whether the treatment supporter received pre-treatment counseling. The topics covered in the FGDs and KIIs included: level of knowledge on TB, distance to a health facility, means of transport, alcohol consumption, perceived TB stigma, debilitating illnesses of caregivers, psychosocial issues, knowledge of TB, and stigma and discrimination, and peer and community support.

### Statistical analysis

#### Qualitative data

We identified major themes that emerge from the data. We used NVivo 11(SQR International, Melbourne, Australia) for systematic data management. The coding of the data proceeded as follows: 1) To identify the major codes, all the transcripts were read and coded by two independent reviewers (JI and SO) who have experience and expertise in mixed-methods research.

The independent coding prevented selective perception and interpretive bias and ensured a rigorous coding process; (2) Codes were then compared, and after resolving any discrepancies, a harmonized codebook was developed; and (3) Using the constant comparison method, we developed codes to capture emergent themes. The analysis continued with the refining of the major themes and codes through discussion between investigators.

#### Quantitative data

The filled data abstraction tool was checked for accuracy and completeness in real-time and then double-entered in Epi-Data version 3.1 [15], with quality control measures such as legal and range values, skip patterns, and alerts. We perform descriptive data analysis where categorical data like sex was summarized using frequencies and percentages, while continuous data like age were summarized using means and standard deviation. In the bivariate analysis, we examined differences in DTT with categorical data using the Chi-square or Fisher’s exact test. For continuous data, the student’s t-test or Wilcoxon-rank sum test was used. Variables with *p* < 0.15 at bivariate analysis were considered statistically significant for multivariate analysis to control for residual confounding. We used a stepwise generalized linear model, excluding variables that did not improve the fit of the regression model based on the log-likelihood to achieve model parsimony. The results were reported as unadjusted odds ratio (OR) and adjusted odds ratio (aOR), with the corresponding 95% confidence interval (CI). The final model showed a good fit namely, a low Akaike Information Criteria (AIC) of 211.5, statistically insignificant Hosmer-Lemeshow Chi-square test (degree of freedom = 8, statistic < 0.01, *p* = 1.000), and a large C-statistics or area under the receiver operating curve of 78.5%.

#### Ethical consideration

The study received ethical approval from Clarke International University Research Ethics Committee (REC number: CLARKE-2020-8). Administrative approval was granted by the Directorate of Public Health and Environment at KCCA. All the respondents provided informed consent after explaining the study purpose, processes, benefits, and potential harms.

## Results

### Study profile

**Fig. 1:**
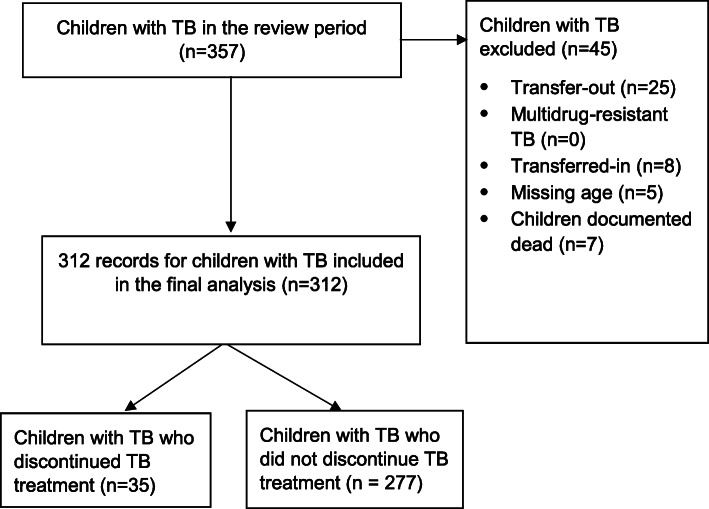
Study profile for discontinuation of tuberculosis treatment among children in the Kampala Capital City Authority health facilities

Between January 2018 and December 2019, there were 357 children diagnosed with and treated for TB across the study sites. Of these children with TB, we excluded 45 for the following reasons: 25 had transferred out, 8 were transferred-in, 5 had missing age, and 7 had died. Overall, we analysed data for 312 children with TB (Fig. 1).

### Participant’s characteristics and DTT in children

Of 312 participants enrolled, 35 (11.2%) had discontinued TB treatment. 125 (40.1%) participants were aged 6–10 years, 203 (65.1%) females, 255 (81.7%) persons with new TB diagnosis, 178 (57.1%) were cases of CD-PTB and 296 (94.9%) received treatment under community-based DOTS (CB-DOTS). We observed statistically significant differences in DTT with regards to the type of person with TB (*p* = 0.018), type of DOTS (*p* = 0.023), TB/HIV co-infection (*p* = 0.039), and receipt of counselling by the caregiver at the time of TB treatment initiation (*p* = 0.042) (Table 1).

**Table 1:** Analysis of participant’s characteristics by DTT in children

Characteristics	Level	Total (***n*** = 312)	DTT		***p***-value
			No (***n*** = 277)	Yes (***n*** = 35)	
Age categories (years)	0–5	75 (24.0)	63 (22.7)	12 (34.3)	0.221
	6–10	125 (40.1)	115 (41.5)	10 (28.6)	
	11–15	112 (35.9)	99 (35.7)	13 (37.1)	
Sex	Female	203 (65.1)	185 (66.8)	18 (51.4)	0.09
	Male	109 (34.9)	92 (33.2)	17 (48.6)	
Type of person with TB	New	255 (81.7)	232 (83.8)	23 (65.7)	0.018
	Retreatment	57 (18.3)	45 (16.2)	12 (34.3)	
TB disease classification	BC-PTB	87 (27.9)	77 (27.8)	10 (28.6)	0.054
	CD-PTB	178 (57.1)	163 (58.8)	15 (42.9)	
	EPTB	47 (15.1)	37 (13.4)	10 (28.6)	
Type of DOTS	FB-DOTS	16 (5.1)	11 (4.0)	5 (14.3)	0.023
	CB-DOTS	296 (94.9)	266 (96.0)	30 (85.7)	
Anti-TB regimen	2RHZE/6HE	100 (32.1)	93 (33.6)	7 (20.0)	0.125
	2RHZE/4RH	212 (67.9)	184 (66.4)	28 (80.0)	
TB/HIV co-infection	No	251 (80.4)	228 (82.3)	23 (65.7)	0.039
	Yes	61 (19.6)	49 (17.7)	12 (34.3)	
Residence	Rural	112 (35.9)	95 (34.3)	17 (48.6)	0.133
	Urban	200 (64.1)	182 (65.7)	18 (51.4)	
Origin of referral	Community	16 (5.1)	13 (4.7)	3 (8.6)	0.404
	Facility	296 (94.9)	264 (95.3)	32 (91.4)	
Phase of treatment	Intensive	256 (82.1)	231 (83.4)	25 (71.4)	0.100
	Continuation	56 (17.9)	46 (16.6)	10 (28.6)	
Treatment supporter marital status	Married	198 (63.5)	176 (63.5)	22 (62.9)	0.151
	Separated	58 (18.6)	48 (17.3)	10 (28.6)	
	Single	56 (17.9)	53 (19.1)	3 (8.6)	
Treatment supporter is employed	No	181 (58.0)	166 (59.9)	15 (42.9)	0.069
	Yes	131 (42.0)	111 (40.1)	20 (57.1)	
Relationship between the treatment supporter and child with TB	Grand parents	45 (14.4)	41 (14.8)	4 (11.4)	0.937
	Biological parents	174 (55.8)	154 (55.6)	20 (57.1)	
	Other relatives	93 (29.8)	82 (29.6)	11 (31.4)	
Treatment supporter received counseling at the time of TB treatment initiation	No	48 (15.4)	38 (13.7)	10 (28.6)	0.042
	Yes	264 (84.6)	239 (86.3)	25 (71.4)	

Note: 1) BC-PTB: Bacteriologically confirmed pulmonary tuberculosis; 2) CB-DOTS: Community-based Directly Observed Therapy Short Course; 3) CD-PTB: Clinically diagnosed pulmonary tuberculosis; 4) FB-DOTS: Facility-based Directly Observed Therapy Short Course; 5) RHZE: Rifampicin, Isoniazid, Pyrazinamide, and Ethambutol

### Reasons for DTT in children

Five sub-themes emerged as reasons for DTT from the qualitative study: 1) no privacy at the healthcare facility to people with TB and their treatment supporters; 2) the disappearance or symptom of TB reduction makes most people with TB discontinue treatment; 3) Poor implementation of CB-DOTS hinders treatment continuation; 4) Insufficient funding prevents the follow-up of lost patients to continue TB treatment, and 5) Frequent stock out of TB drugs frustrates people with TB from continuing with treatment (Table 2).

**Table 2:** Summary of reasons for DTT in children

Participant quotations	Reason for DTT
*“Let me say again that the working space is very small -the buildings … they are not enough. And when you’re counseling for HIV, you don’t have separate rooms … we sit here in this room. The same room, we are using for clinical, other clinical services … No privacy”*. (KII, Health worker)	No privacy at the healthcare facility to people with TB and their treatment supporters
*“We see over a hundred patients a day. So, in bad weather like this, they have nowhere to sit. So, you see them scattered there … to crowd on that veranda”* and: *“There is no privacy there. If you put a counselor there, no, it doesn’t work because everybody enters there”.* “(KII, Health worker)	
“*Getting well and disappearance of symptoms is major cause of interruption or default from treatment also the inconvenience caused by busy working caregivers in formal and informal setting”* (FGD, Treatment supporters).	The disappearance or reduction in symptoms of TB makes most persons with TB discontinue treatment
*“TB patients are supposed to be supported at home, that is DOTS- he is supposed to be observed by his treatment supporter when swallowing tablets daily and then the health worker is supposed to go and take the medicines every 2 weeks. But actually, the coverage [of DOTS] is low because of lack of resources.* (KII, Health worker)	Poor implementation of community-based DOTS hinders treatment continuation
*We have no funds to support people to go down there [to the residence/villages/zones]. The Ministry of Health doesn’t actually have money to give [to the National TB Program] to support DOTS. The divisions receive Primary Health Care funds, but they are not adequate to support [DOTS] they [the government] are not prioritizing TB DOTS strategy.”* (KII, Health worker) “*Often clinic appointment may come when you don’t have money for transport and if the child is feeling ok, you may not be bothered to pick medicines” also lack of support and proper counselling from the health workers may lead to treatment discontinuation*” (FGD, Treatment supporters).	Insufficient funding prevents the follow-up of lost patients to continue TB treatment by health workers and prevents patient’s returns to per clinic appointment.
*“Without the capacity to follow up with patients when they don’t visit to the health center to collect their drugs, health care workers are not able to determine whether TB patients have dropped off, defaulted, died, or transferred to another health center. It is not uncommon for patients to decide to be treated at a different health center, because of the way health centers are located, however no mechanism is in place to track those patients.”* (KII, Health worker)	
“*Sometimes there’s frustrations. There are no medications. It’s just very difficult to have to work. It’s difficult, even when you want to support a patient, sometimes you don’t have … it’s a bit difficult, but we try to improvise sometimes and make ends meet but this usually disappoints our patients and may not come back [meaning that people with TB do not continue with treatment]*” *.* (KII, Health worker)	Frequent stock-out of TB drugs frustrates people with TB from continuing with treatment**.**
*“Sometimes we run short of drugs here.... When we run short of drugs, you put requests in time, these drugs come late, and I mean, it interrupts treatment [meaning discontinuation of TB treatment]. You find somebody missing out on treatment for a month. It’s a very big challenge*”.(KII, Health worker)	

### Factors associated with DTT in children

In the unadjusted analysis (Table 3), we observed CB-DOTS (OR, 0.25; 95% CI, 0.08–0.83) and counselling of treatment supporters at the time of treatment initiation (OR, 0.40; 95% CI, 0.18–0.93) as associated with a lower likelihood of DTT. DTT was more likely among retreatment TB cases compared to the new cases of TB (OR, 2.69; 95% CI, 1.22–5.72) and among children with TB/HIV compared to HIV-negative children with TB (OR, 2.43; 95% CI, 1.10–5.14).

**Table 3:** Factors associated with DTT in children at unadjusted and adjusted analysis using generalized linear modelling

		Univariable	Multivariable
Characteristics	Level	OR (95% CI)	aOR (95% CI)
Age categories (years)	0–5	1	
	6–10	0.46 (0.18–1.12)	
	11–15	0.69 (0.29–1.62)	
Sex	Female	1	
	Male	1.90 (0.93–3.87)	
Type of person with TB	New	1	
	Retreatment	2.69* (1.22–5.72)	
Category of TB disease	PBC	1	
	PCD	0.71 (0.31–1.70)	
	EPTB	2.08 (0.79–5.50)	
Type of DOTS	Facility	1	1
	Community	0.25* (0.08–0.83)	0.33 (0.10–1.16)
Regimen	2RHZE/6HE	1	
	2RHZE/4RH	2.02 (0.90–5.18)	
TB/HIV co-infection	No	1	
	Yes	2.43* (1.10–5.14)	
Residence	Rural	1	
	Urban	0.55 (0.27–1.13)	
Origin of referral	Community	1	
	Facility	0.53 (0.16–2.38)	
Phase of treatment	Intensive	1	1
	Continuation	2.01 (0.87–4.36)	5.22** (1.76–17.52)
Treatment supporter marital status	Married	1	
	Separated	1.67 (0.71–3.68)	
	Single	0.45 (0.10–1.37)	
Treatment supporter is employed	No	1	1
	Yes	1.99 (0.98–4.12)	3.60* (1.34–11.38)
Relationship between the treatment supporter and child	Grandparents	1	
	Biological parents	1.33 (0.47–4.76)	
	Other relatives	1.37 (0.44–5.20)	
Treatment supporter received counseling at the time of TB treatment initiation	No	1	
	Yes	0.40* (0.18–0.93)	

Note: 1) BC-PTB: Bacteriologically confirmed pulmonary tuberculosis; 2) CB-DOTS: Community-based Directly Observed Therapy Short Course; 3) CD-PTB: Clinically diagnosed pulmonary tuberculosis; 4) FB-DOTS: Facility-based Directly Observed Therapy Short Course; 5) RHZE: Rifampicin, Isoniazid, Pyrazinamide, and Ethambutol; 6) 2) Statistical significance codes at 5%: *** *p* < 0.001, ** *p* < 0.01, * *p* < 0.05

DTT was not associated with being in the age categories of 6–10 years (OR, 0.46; 95% CI, 0.18–1.12) or 11–15 years (OR, 0.69; 95% CI, 0.29–1.62), male sex (OR, 1.90; 95% CI, 0.93–3.87), being a PCD (OR, 0.71; 95% CI, 0.31–1.70) or EPTB case (OR, 2.08; 95% CI, 0.79–5.50), treatment with 2RHZE/4RH (OR, 2.02; 95% CI, 0.90–5.18), being an urban resident (OR, 0.55; 95% CI, 0.27–1.13), referral from health facility (OR, 0.53; 95% CI, 0.16–2.38) and the continuation phase of TB treatment (OR, 2.01; 95% CI, 0.87–4.36). Concerning treatment supporter factors, DTT was not associated with being single (OR, 0.45; 95% CI, 0.10–1.37) or separated (OR, 1.67; 95% CI, 0.71–3.68), employed (OR, 1.99; 95% CI, 0.98–4.12), biological parent (OR, 1.33; 95% CI, 0.47–4.76) or other relative (OR, 1.37; 95% CI, 0.44–5.20).

In the adjusted analysis results (Table 3), DTT was more likely during the continuation phase of TB treatment compared to the intensive phase (aOR, 5.22; 95% CI, 1.76–17.52) and when the treatment supporter was employed compared to when the treatment supporter was unemployed (aOR, 3.60; 95% CI, 1.34–11.38). CB-DOTS showed a tendency toward lower DTT compared to facility-based DOTS (FB-DOTS) but the result remained statistically insignificance (aOR, 0.33; 95% CI, 0.10–1.16).

## Discussion

The focus of this study is on DTT among children in Kampala Uganda. Our data show that for every ten children with TB, at least one discontinues TB treatment. The commonest reasons for DTT include lack of privacy at healthcare facilities for children with TB and their treatment supporters, the disappearance of TB symptoms following treatment initiation, poor implementation of the CB-DOTS, insufficient funding to the TB program, and frequent stock-outs of TB drugs. DTT is more likely in the continuation phase of TB treatment and when the treatment supporter is employed. We observed a tendency towards a lower likelihood of DTT when treatment is under CB-DOTS strategy compared to FB-DOTS strategy although the association did not reach statistical significance.

Few studies have looked at DTT among children in Uganda and the sub-Saharan African region. However, the prevalence of DTT in the present study is comparable to that in a previous study in western Uganda [16] but slightly more than half the prevalence of DTT in a Nigerian study [17]. The differences in DTT might be attributable to the study population. The focus of the present study is on children with TB, who mostly depend on adults for continuity of care while the study in Nigeria and Uganda focused on adults with TB who often make independent decisions regarding their health. In general, the high prevalence of DTT among children should be a central concern for the TB control program as it potentially leads to severe forms of TB, TB-related complications, and an increased risk of morbidity and mortality among children with TB. Lack of privacy to people with TB and treatment supporters at the healthcare facilities, the disappearance or reduction in the symptoms of TB symptoms after treatment initiation, the poor implementation of CB-DOTS strategy, insufficient funding that prevents the follow-up of lost patients, and frequent stock-outs of TB drugs were reasons for DTT. To the best of our knowledge, these factors are not unique.

A previous study in eastern Uganda reports that inadequate financing of the TB control program and deficiencies in the implementation of the CB-DOTS strategy is associated with suboptimal treatment success [18]. The study further showed that the disappearance of symptoms of TB especially after treatment initiation results in DTT [18]. Physical and economic barriers have been reported in a previous study as one of the reasons for not completing required sputum smear monitoring visits [19], emphasizing that people with TB face several barriers along the pathway to continuing with treatment. These reasons underscore the importance of strengthening the health system in ensuring adequate financing and sustainable supply of drugs and equipment, among others [20]. The findings further highlight the importance of involving persons with TB and their treatment supporters in TB care [21].

The finding that DTT is more likely in the continuation phase compared to the intensive phase is consistent with previous studies in sub-Saharan Africa [17, 22]. This finding could be explained by several plausible reasons. In the intensive phase of TB treatment, people with TB and their treatment supporters are required to visit TB clinics every 2 weeks to receive health education and treatment. In the continuation phase, the visits occur monthly. This implies that people with TB and their treatment supporters have less contact with health workers in the continuation phase compared to the intensive phase. This possibly might have contributed to laxity among the treatment supporters in helping the children with TB to continue with treatment. Another plausible explanation is that in the continuation phase, most of the symptoms of TB tend to resolve and people with TB feel much better and stronger. The urge to discontinue TB treatment is thus potentially high. One study in eastern Uganda reports that people with TB tended to discontinue treatment when they no longer have symptoms of TB or when they felt better [19]. This finding implies that there is a need to continue providing key messages about TB treatment such as the benefits of treatment completion and the risks associated with DTT in the continuation phase.

There is also a need to explore additional options for the effective delivery of TB treatment in the continuation phase. For example, prospective studies should explore the feasibility and acceptability of multi-month dispensing of TB drugs and the effect on treatment outcomes through implementation science research.

Our study shows that DTT is more likely among treatment supporters with employment compared to the unemployed consistent. No previous study in our setting and elsewhere report similar findings. However, the most socially plausible explanation might be that employed treatment supporters face difficulties in getting time off from their work to travel to TB clinics on regular basis. This potentially leads to DTT.

We found a tendency towards a lower likelihood of DTT for participants under the CB-DOTS strategy compared to the FB-DOTS strategy, which is not surprising. Previous systematic reviews and meta-analyses indicate that the CB-DOTS strategy is associated with favorable treatment outcomes, namely high treatment completion and success rates, and lower mortality and loss to follow-up [23–25]. This is because the CB-DOTS strategy improves access to TB treatment since drug refills are done within the community where people with TB and their treatment supporters live and work. In other words, the CB-DOTS strategy overcomes physical and economic barriers that hinder access to TB care and that impact negatively on TB treatment outcomes. Although further research is needed to understand this finding, it could potentially be attributable to the smaller number of participants in the FB-DOTS compared to the CB-DOTS group or disease severity, with those FB-DOTS being sicker than those on CB-DOTS hence at higher risk of adverse outcomes including death. Our finding thus highlights the importance of strengthening the implementation of the CB-DOTS strategy.

Our study has several strengths. This study is among the first few studies to determine DTT among children in Uganda and sub-Saharan Africa. The use of qualitative data to underscore reasons for DTT from the treatment supporter and healthcare provider perspectives further strengthens the quantitative evidence. However, there are limitations to consider. Our study was conducted among children in an urban setting so the findings might not be generalized to a rural setting where access to healthcare is problematic due to longer travel distances. Since we analyzed secondary data, there is a possibility of missing data entries in the TB registers. The study sample size had merely a 45% chance of finding a statistically significant difference assuming DTT is the same in the continuation phase. Our study thus is underpowered to detect a statistically significant difference. The purposive selection of participants could have possibly biased the findings. Also, the lack of individual data from each of the DTT patients to know their reasons is another limitation.

## Conclusions and recommendations

This study shows that at least 10% of children with TB discontinue treatment and this might exacerbate TB morbidity and mortality. The main reasons for DTT include lack of privacy at healthcare facilities for people with TB and their treatment supporters, the disappearance or reduction in symptoms of TB symptoms after treatment initiation, poor implementation of CB-DOTS strategy, insufficient funding which prevents the healthcare system from initiating and conducting follow-ups for lost patients, and frequent stock-outs of TB drugs which frustrates people with TB from continuing with treatment. DTT is more likely in the continuation phase of TB treatment and when the treatment supporter is employed. To mitigate DTT, we recommend that healthcare providers should ensure children with TB and their treatment supporters are accorded privacy during service provision and provide more information about TB symptom resolution and treatment duration versus the need to complete treatment.

The district and national TB control programs should address gaps in funding to TB care, the supply of TB drugs, and the implementation of the CB-DOTS strategy.

## Data Availability

To protect participant’s anonymity, data will be shared at reasonable request. The contact person is: Stephen Okoboi: okoboi25@gmail.com. A statement to confirm that all methods were carried out in accordance with relevant guidelines and regulations.

## Abbreviations

CB-DOTS: Community-based Directly Observed Therapy Short course
FB-DOTS: Facility-based Directly Observed Therapy Short course
DTT: Discontinuation of tuberculosis treatment
